# Supplementary material for: Col4a3-/- Mice on Balb/C Background Have Less Severe Cardiorespiratory Phenotype and SGLT2 Over-Expression Compared to 129x1/SvJ and C57Bl/6 Backgrounds
Source: Int J Mol Sci. 2022 Jun 15;23(12):6674. doi: 10.3390/ijms23126674 (PMC9223785; doi:10.3390/ijms23126674)
Supplement: Supplementary file 1 [file ijms-23-06674-s001.zip › ijms-1762702-supplementary.pdf]

**Table S1.** Echocardiography parameters of Pulse-wave doppler (PWD) of pulmonary valve, ascending aorta and diaphragm ultrasonography in male mice from 129J, Balb/C and C57Bl/6 background

| **Echocardiography parameters** | **129J 8 weeks old** | | **Balb/C 8 weeks old** | | **C57Bl/6 20 weeks old** | |
|---|---|---|---|---|---|---|
| **Pulmonary valve** | | | | | | |
| | **WT (n=13)** | ***Col4a3***$^{-/-}$**(n=14)** | **WT (n=12)** | ***Col4a3***$^{-/-}$ ***(n=9)*** | **WT (n=11)** | ***Col4a3***$^{-/-}$***(n=9)*** |
| PAT (ms) | 20.53 ± 3.63 | 18.99 ± 4.6 | 18.73 ± 2.74 | 18.55 ± 3.38 | 18.59 ± 3.58 | 19.78 ± 3.29 |
| PET (ms) | 61. 50 ± 2.83 | 56.35 ± 9.10 | 62.48 ± 8.11 | 60.77 ± 4.11 | 53.51 ± 4.13 | 55.80 ± 2.80 |
| PV Peak Vel (mm/s) | -689±118.23$^{€€}$ | -609.59±105.58 | -529.85 ± 87.80$^{†}$ | -576.33 ± 90.07 | -689.88 ± 162.09 | -669.29 ± 83.74 |
| PV VTI (mm) | 30.1 ± 5.06 | 23.39 ± 7.82* | 23.57 ± 4.29 | 25.65 ± 3.91 | 26.91 ± 5.54 | 26.06 ± 3.33 |
| Mean Vel (PV VTI, mm/s) | -415.8 ± 68.5 | -358.4 ± 63.65 | -334.4 ± 57.15 | -365.5 ± 61.37 | -436.8 ± 103.3 | -395.7 ± 73.6 |
| Mean Grad (PV VTI, mmHg) | 0.71 ± 0.23 | 0.53 ± 0.19 | 0.46 ± 0.15 | 0.55 ± 0.20 | 0.80 ± 0.36 | 0.65 ± 0.23 |
| Peak Vel (PV VTI, mm/s) | -705.1 ± 120.4 | -609.4 ± 107 | -539.9 ± 91.52$^{€,†}$ | -588.1 ± 98.58 | -697.2 ± 161.9 | -685.6 ± 72.03 |
| Peak Grad (PV VTI, mmHg) | 2.04 ± 0.67 | 1.53 ± 0.54 | 1.20 ± 0.38$^{€,†}$ | 1.42 ± 0.49 | 2.04 ± 0.89 | 1.9 ± 0.39 |
| MPAP (common, mmHg) | 69.6 ± 1.70 | 70.46 ± 2.06 | 70.57 ± 1.24 | 70.65 ± 1.52 | 70.64 ± 1.61 | 70.1 ± 1.48 |
| MPAP (PAT < 120 ms, mmHg) | 76.97 ± 2.24 | 78.23 ± 2.83 | 78.39 ± 1.70 | 78.5 ± 2.1 | 78.48 ± 2.22 | 77.73 ± 2.04 |
| PAT/PET | 0.33 ± 0.057 | 0.33 ± 0.047 | 0.30 ± 0.04 | 0.31 ±0.05 | 0.34 ± 0.05 | 0.35 ± 0.05 |
| PV Peak Pressure (mmHg) | 1.96 ± 0.66 | 1.53 ± 0.53 | 1.15 ± 0.36$^{€,†}$ | 1.36 ± 0.44 | 1.99 ± 0.88 | 1.82 ± 0.45 |
| **Ascending Aorta** | | | | | | |
| | **WT (n=13)** | ***Col4a3***$^{-/-}$**(n=15)** | **WT (n=12)** | ***Col4a3***$^{-/-}$ ***(n=9)*** | **WT (n=11)** | ***Col4a3***$^{-/-}$***(n=10)*** |
| AET (ms) | 50.31 ± 3.12 | 45.67 ± 5.0$^{€€, ¥¥}$ | 56.32 ± 6.27 | 56.67 ± 7.33 | 51.50 ± 6.39 | 55.03 ± 9.15 |
| Aortic valve Peak Velocity (mm/s) | 817.38 ± 226.07$^{€€€}$ | 893.92±127.13$^{€€€}$ | 522.97±127.20 | 555.87±139.26 | 613.28±176.67 | 661.54±199.93¥ |
| AV Peak Pressure (mmHg) | 2.86 ± 1.45$^{€€€,¥}$ | 3.26 ± 0.91$^{€€€,¥}$ | 1.15 ±0.48 | 1.31 ± 0.69 | 1.62 ± 0.85 | 1.89 ± 1.14 |
| **Diaphragm ultrasonography** | | | | | | |
| | **WT (n=13)** | ***Col4a3***$^{-/-}$**(n=15)** | **WT (n=12)** | ***Col4a3***$^{-/-}$ ***(n=9)*** | **WT (n=11)** | ***Col4a3***$^{-/-}$***(n=10)*** |
| Diaphragm movement (in mm) | 0.83 ± 0.10 | 0.58 ± 0.065 | 0.95 ± 0.12 | 0.89 ± 0.16 | 1.07 ± 0.27 | 0.9 ± 0.36 |

PAT: Pulmonary Acceleration Time, PET: Pulmonary Ejection Time, PV: Pulmonary Valve, VTI: Velocity Time Integral, Vel: Velocity, Grad: Gradient, MPAP: Mean Pulmonary Artery Pressure, AET: Aortic Ejection Time, AV: Aortic Valve, ms: milliseconds, s: seconds, mm: millimeters. Values are mean ± SD. *p<0.05, **p<0.01, ***p<0.001 and ****p<0.0001 compared Col4a3-/- with their respective control (same strain), †P<0.05, ††p<0.01, †††p<0.001, ††††p<0.0001 differences between Balb/C strain and C57Bl/6 strain (WT vs WT and Col4a3-/- vs Col4a3-/-), €p<0.05, €€p<0.01, €€€p<0.001, €€€€p<0.0001 differences between Balb/C strain and 129J (WT vs WT and Col4a3-/- vs Col4a3-/-), ¥P<0.05, , ¥¥p<0.01, ¥¥¥ p<0.001, ¥¥¥¥p<0.0001 difference between 129J and C57Bl/6 (WT Vs WT and Col4a3-/- vs Col4a3-/-).

**Table S2.** Echocardiography parameters of Pulse-wave doppler (PWD) of pulmonary valve, ascending aorta and diaphragm ultrasonography in female mice from 129J, Balb/C and C57Bl/6 background

| Echocardiography parameters | 129J 8 weeks old | | Balb/C 8 weeks old | | C57Bl/6 20 weeks old | |
|---|---|---|---|---|---|---|
| **Pulmonary valve** | | | | | | |
| | **WT (n=13)** | ***Col4a3**$^{-/-}$**(n=16)** | **WT (n=12)** | ***Col4a3**$^{-/-}$ ***(n=14)*** | **WT (n=14)** | ***Col4a3**$^{-/-}$***(n=10)*** |
| PAT(ms) | 18.23 ± 2.31 | 20.69 ± 3.40 | 18.43 ± 4.28 | 18.06 ± 3.85 | 19.50 ± 2.5 | 20.36 ± 3.78 |
| PET(ms) | 58.99 ± 4.93 | 59.64 ± 6.72 | 59.73 ± 4.65 | 65.57 ± 7.28$^{\dagger\dagger\dagger}$ | 54.82 ± 3.13 | 54.44 ± 2.82 |
| PV Peak Vel (mm/s) | -792.67±166.13$^{\text{€€}}$ | -723.34±156.9$^{\text{€€}}$ | -591.48±134.04 | -536.61±125.96 | -651.96 ± 90.22 | -662.36 ±130.35 |
| PV VTI (mm) | 31.13 ± 6.44 | 28.98 ± 5.31 | 26.38 ± 5.25 | 24.79 ± 5.89 | 25.32 ± 3.81 | 24.14 ± 4.67 |
| Mean Vel (PV VTI, mm/s) | -468.7 ± 96.99 | -430.4 ± 88.2$^{\text{€}}$ | -377.4 ± 81.09 | -332.4 ± 70.65 | -404.5 ± 62.05 | -410.8 ± 69.39 |
| Mean Grad (PV VTI, mmHg) | 0.91 ± 0.37$^{\text{€}}$ | 0.77 ± 0.32$^{\text{€}}$ | 0.59 ± 0.24 | 0.46 ± 0.18 | 0.67 ± 0.2 | 0.69 ± 0.22 |
| Peak Vel (PV VTI, mm/s) | -797.3 ± 170.5$^{\text{€}}$ | -727.3 ± 157.9$^{\text{€€}}$ | -609 ± 118.8 | -544.6 ± 122.3 | -666.2 ± 97.74 | -672.3 ± 130 |
| Peak Grad (PV VTI, mmHg) | 2.65 ± 1.11$^{\text{€€}}$ | 2.21 ± 0.96$^{\text{€}}$ | 1.53 ± 0.56 | 1.24 ± 0.51 | 1.81 ± 0.50 | 1.87 ± 0.69 |
| MPAP (common, mmHg) | 70.8 ± 1.04 | 69.69 ± 1.53 | 70.71 ± 1.92 | 70.88 ± 1.73 | 70.22 ± 1.12 | 69.84 ± 1.70 |
| MPAP (PAT < 120 ms, mmHg) | 78.7 ± 1.43 | 77.17 ± 2.11 | 78.58 ± 2.65 | 78.81 ± 2.39 | 77.91 ± 1.55 | 77.38 ± 2.34 |
| PAT/PET | 0.31 ± 0.035 | 0.35 ± 0.04 $^{\text{€€}}$ | 0.31 ± 0.07 | 0.28 ± 0.06$^{\dagger\dagger\dagger}$ | 0.36 ± 0.036 | 0.375 ± 0.071 |
| PV Peak Pressure (mmHg) | 2.62 ± 1.07$^{\text{€€, ¥}}$ | 2.18 ± 0.95$^{\text{€€}}$ | 1.47 ± 0.6 | 1.21 ± 0.52 | 1.73 ± 0.45 | 1.82 ± 0.68 |
| **Ascending Aorta** | | | | | | |
| | **WT (n=13)** | ***Col4a3**$^{-/-}$**(n=16)** | **WT (n=12)** | ***Col4a3**$^{-/-}$ ***(n=14)*** | **WT (n=14)** | ***Col4a3**$^{-/-}$***(n=10)*** |
| AET (ms) | 51.37 ± 3.08$^{\text{€}}$ | 49.59 ± 2.44$^{\text{€€}}$ | 57.63 ± 4.82 | 55.98 ± 4.51 | 52.72 ± 3.09 | 52.76 ± 8.04 |
| Aortic valve Peak Velocity (mm/s) | 10009 ± 216.11$^{\text{€€€}}$ | 959.1 ± 156.6$^{\text{€€€}}$ | 504.36 ± 159.3 | 452.14 ± 138.79 | 620.24 ± 134.54$^{\text{¥}}$ | 557.13 ± 136.99$^{\text{¥¥}}$ |
| AV Peak Pressure (mmHg) | 4.01± 1.59$^{\text{€€€€}}$ | 3.77 ± 1.21$^{\text{€€€€}}$ | 1.11 ± 0.79 | 0.76 ± 0.31 | 1.61 ± 0.69$^{\text{¥¥¥¥}}$ | 1.16 ± 0.41$^{\text{¥¥¥¥}}$ |
| **Diaphragm ultrasonography** | | | | | | |
| | **WT (n=13)** | ***Col4a3**$^{-/-}$**(n=15)** | **WT (n=12)** | ***Col4a3**$^{-/-}$ ***(n=9)*** | **WT (n=11)** | ***Col4a3**$^{-/-}$***(n=10)*** |
| Diaphragm movement (in mm) | 0.86 ± 0.17 | 0.75 ± 0.28 | 0.95 ± 0.25 | 0.86 ± 0.26 | 0.98 ± 0.26 | 0.79 ± 0.2 |

PAT: Pulmonary Acceleration Time, PET: Pulmonary Ejection Time, PV: Pulmonary Valve, VTI: Velocity Time Integral, Vel: Velocity, Grad: Gradient, MPAP: Mean Pulmonary Artery Pressure, AET: Aortic Ejection Time, AV: Aortic Valve, ms: milliseconds, s: seconds, mm: millimeters. Values are mean ± SD. *p<0.05, **p<0.01, ***p<0.001 and ****p<0.0001 compared Col4a3-/- with their respective control (same strain), †p<0.05, ††p<0.01, †††p<0.001, ††††p<0.0001 differences between Balb/C strain and C57Bl/6 strain (WT vs WT and Col4a3-/- vs Col4a3-/-), €p<0.05, €€p<0.01, €€€p<0.001, €€€€p<0.0001 differences between Balb/C strain and 129J (WT vs WT and Col4a3-/- vs Col4a3-/-), ¥P<0.05, , ¥¥p<0.01, ¥¥¥ p<0.001, ¥¥¥¥p<0.0001 difference between 129J and C57Bl/6 (WT Vs WT and Col4a3-/- vs Col4a3-/-).

**Table S3.** Heart and lung gravimetric data for male and females mice from 129J, Balb/C and C57Bl/6 background

| Gravimetric parameters | 129J 8 weeks old | | Balb/C 8 weeks old | | C57Bl/6 20 weeks old | |
|---|---|---|---|---|---|---|
| **Male** | | | | | | |
| | **WT (n=17)** | ***Col4a3*$^{-/-}$(n=18)** | **WT (n=13)** | ***Col4a3*$^{-/-}$ *(n=5)*** | **WT (n=13-15)** | ***Col4a3*$^{-/-}$*(n=9-10)*** |
| BW (g) | 21.81 ± 2.09 | 17.62 ± 2.36$^{****}$ | 23.25 ± 3.22$^{\dagger\dagger\dagger}$ | 19.89 ± 0.66$^{*,\dagger\dagger\dagger\dagger}$ | 27.56 ± 2.32$^{\text{¥¥¥¥}}$ | 26.11 ± 1.93$^{\text{¥¥¥¥}}$ |
| HW (mg) | 115.7 ± 16.64 | 115.6 ± 18.8 | 124.1 ± 20.7 | 117.9 ± 21.77 | 116.8 ± 16.57 | 128.1 ± 16.18 |
| Tibia length (cm) | 1.73 ± 0.08 | 1.73 ± 0.07 | 1.71 ± 0.07 | 1.64 ± 0.05$^{\dagger\dagger}$ | 1.75 ± 0.06 | 1.78 ± 0.04 |
| HW/BW (mg/g) | 5.31 ± 0.59 | 6.6 ± 0.87$^{***}$ | 5.3 ± 0.49 | 6.2 ± 1.04 | 5.50 ± 0.52 | 5.41 ± 0.68$^{\text{¥¥¥}}$ |
| HW/TL(mg/cm) | 66.85 ± 8.86$^{\text{¥¥¥}}$ | 66.74 ± 11.03 | 72.44 ± 9.67$^{\dagger}$ | 71.89 ± 11.70 | 85.87 ± 14.29 | 79.25 ± 9.67 |
| LW(Wet) (mg) | 127.1 ± 15.88$^{\text{¥¥¥}}$ | 131.6 ± 25.5$^{\text{¥¥}}$ | 149.9 ± 17.67 | 160 ± 43.98 | 170.3 ± 27.12 | 178.3 ± 37.76 |
| LW/BW (mg/g) | 6.11 ± 1.04 | 7.47 ± 0.99$^{**}$ | 6.54 ± 1.12 | 8.47 ± 2.57 | 6.21 ± 1.31 | 6.90 ± 1.59 |
| LW/TL (mg/cm) | 76.44 ± 10.84$^{\text{¥¥¥}}$ | 75.99 ± 14.83$^{\text{¥¥}}$ | 87.75 ± 9.75 | 97.81 ± 27.34 | 97.32 ± 16.95 | 100.31 ± 21.03 |
| **Female** | | | | | | |
| | **WT (n=19)** | ***Col4a3*$^{-/-}$(n=13-14)** | **WT (n=11-13)** | ***Col4a3*$^{-/-}$ *(n=10-12)*** | **WT (n=13-14)** | ***Col4a3*$^{-/-}$*(n=11)*** |
| BW (g) | 17.23 ± 1.86$^{\text{¥¥¥}}$ | 15.58 ± 1.71$^{\text{¥¥¥}}$ | 17.74 ± 2.10$^{\dagger\dagger\dagger}$ | 16.07 ± 0.73$^{\dagger\dagger\dagger}$ | 21.39 ± 1.81 | 20.94 ± 1.56 |
| HW (mg) | 84.75 ± 13.21$^{\text{¥¥¥}}$ | 92.81 ± 14.98$^{\text{¥¥¥},\dagger}$ | 103.2 ±10.77 | 101.8 ± 10.27 | 116.8 ± 16.57 | 128.1 ± 16.18 |
| Tibia length (cm) | 1.72 ± 0.11$^{\text{€}}$ | 1.64 ± 0.08 $^{\text{¥¥}}$ | 1.63 ± 0.07$^{\dagger\dagger\dagger\dagger}$ | 1.62 ± 0.037 $^{\dagger\dagger\dagger\dagger}$ | 1.78 ± 0.07 | 1.76 ± 0.06 |
| HW/BW (mg/g) | 5.0 ± 0.59 $^{\text{€}}$ | 5.89 ± 0.76$^{*}$ | 5.82 ± 0.64 | 6.22 ± 0.5 | 5.38 ± 0.80 | 6.16 ± 1.02 |
| HW/TL(mg/cm) | 49.26 ± 6.9$^{\text{€€€€,¥¥¥¥}}$ | 56.34 ± 7.46$^{\text{¥¥¥¥}}$ | 63.25 ± 5.8 | 62.78 ± 6.25$^{\dagger}$ | 64.74 ± 8.35 | 72.81 ± 8.9 |
| LW (Wet) (mg) | 119.4 ± 22.08$^{\text{¥¥}}$ | 101.1 ± 15.83$^{\text{¥¥¥,€€}}$ | 143 ± 37.15 | 151 ± 49.86 | 167.2 ± 45.28 | 167.4 ± 40.28 |
| LW/BW (mg/g) | 6.96 ± 1.22 | 7.22 ± 1.37 | 7.97 ± 1.96 | 8.6 ± 1.69 | 7.89 ± 2.12 | 7.95 ±1.66 |
| LW/TL (mg/cm) | 69.46 ± 12.44$^{\text{¥¥}}$ | 68.69 ± 11.37$^{\text{¥}}$ | 86.62 ± 20.96 | 84.55 ± 16.26 | 94.89 ± 23.33 | 95.15 ± 22.51 |

BW: Body weight, HW: Heart weight, TL: tibia length, LW: lung weight, W: wet, D: dry, mg: miligrams, g: grams, cm: centimeters. Values are mean ± SD. *p<0.05, **p<0.01, ***p<0.001 and ****p<0.0001 compared Col4a3-/- with their respective control (same strain), †p<0.05, ††p<0.01, †††p<0.001, ††††p<0.0001 differences between Balb/C strain and C57Bl/6 strain (WT Vs WT and *Col4a3*$^{-/-}$ vs *Col4a3*$^{-/-}$), €p<0.05, €€p<0.01, €€€p<0.001, €€€€p<0.0001 differences between Balb/C strain and 129J (WT vs WT and *Col4a3*$^{-/-}$ vs Col4a3$^{-/-}$), ¥P<0.05, , ¥¥p<0.01, ¥¥¥ p<0.001, ¥¥¥¥p<0.0001 difference between 129J and C57Bl/6 (WT Vs WT and *Col4a3*$^{-/-}$ vs *Col4a3*$^{-/-}$).

**Table S4.** Pressure-volume analyses and hemodynamic data of male mice from 129J, Balb/C and C57Bl/6 background by ventricular catheterization

| Echocardiography parameters | 129J 8 weeks old | | Balb/C 8 weeks old | | C57Bl/6 20 weeks old | |
|---|---|---|---|---|---|---|
| **Pulmonary valve** | | | | | | |
| | **WT (n=7)** | ***Col4a3*$^{-/-}$(n=6)** | **WT (n=5)** | ***Col4a3*$^{-/-}$ *(n=3)*** | **WT (n=6)** | ***Col4a3*$^{-/-}$*(n=5)*** |
| ESPVR Slope | 2.44 ± 0.7 | 3.6 ± 1.77 | 1.26 ± 0.36 | 3.3 ± 1.63$^{*}$ | 1.35 ± 0.62 | 2.48 ± 0.4$^{**}$ |
| EDPVR Slope | 0.10 ± 0.029 | 0.25 ± 0.16$^{*,*}$ | 0.19 ± 0.105 | 0.12 ± 0.065 | 0.099 ± 0.033 | 107 ± 0.022 |
| Stroke Work (mmHg×μL) | 2150 ± 289.7 | 1462 ± 553.5$^{*}$ | 1411±177.6$^{\text{€, ¥¥¥}}$ | 1679 ± 376.7 | 2707 ±447 | 1994 ±252.8$^{*}$ |
| Cardiac Output (μL/min) | 11717±952.2 | 7388 ± 2219$^{***,*}$ | 11254 ± 1307 | 11137 ± 2658 | 19833 ± 4255$^{\text{¥¥¥},\dagger\dagger\dagger\dagger}$ | 14160 ±2625$^{*,*,\text{¥¥¥}}$ |
| Stroke Volume (μL) | 25.32 ± 1.96 | 16.42 ± 5.03$^{***,*}$ | 23.69 ± 2.88 | 26.55 ± 6.1 | 38.83 ± 8.51$^{\text{¥¥¥},\dagger\dagger\dagger}$ | 29.63 ± 4.13$^{\text{¥¥}}$ |
| Volume Maximum (μL) | 50.15± 8.11 | 48.8 ± 10.52 | 47.17 ± 8.82 | 49.3 ± 8.68 | 74.34 ± 18.96$^{\text{¥¥},\dagger\dagger}$ | 65.63 ± 7.48 |

| | | | | | | |
|---|---|---|---|---|---|---|
| Volume Minimum (μL) | 22.64 ± 8.11 | 31.03 ± 9.20 | 23.47 ± 6.13 | 22.74 ± 3.13 | 35.51 ± 16.96 | 36 ± 9.16 |
| End-systolic Volume (μL) | 25.46 ± 9.4 | 33.91 ± 9.67 | 29.07 ± 8.47 | 31.21 ± 4.3 | 44.34 ± 15.76¥ | 42.6 ± 11.82 |
| End-diastolic Volume (μL) | 48.61 ± 8.80 | 47.16 ± 10.48 | 46.1 ± 8.10 | 47.35 ± 7.72 | 73.06 ± 19.36¥¥, †† | 63.7 ± 7.86 |
| Pressure Maximum (mmHg) | 93.12 ± 5.92 | 97.17 ± 10.11€€ | 84.17 ± 7.56 | 83.26 ± 6.75 | 93.67 ± 7.64 | 83.94 ± 10.99 |
| Pressure minimum (mmHg) | 2.27 ± 0.88€,¥ | 2.82 ± 2.23 | 8.30 ± 4.23 | 10.7 ± 5.68 | 8.32 ± 4.46 | 4.04 ± 2.56 |
| Mean Pressure (mmHg) | 36.47 ± 2.81 | 35.41 ± 7.34 | 41.2 ± 10.59 | 36.76 ± 6.8 | 43.72 ± 6.92 | 33.16 ± 8.29* |
| Pressure Development (mmHg) | 90.85 ± 6.48 | 94.34 ± 9.25 | 75.14 ± 3.81€ | 69.9 ± 1.07€€ | 85.36 ± 8.23 | 79.9 ± 9.51¥ |
| End-systolic Pressure (mmHg) | 85.93 ± 5.82 | 93.03 ± 9.8 | 83.36 ± 7.47 | 80.57 ± 11.32 | 93.04 ± 7.76 | 71.45 ± 20.88*,*,¥ |
| End-diastolic Pressure (mmHg) | 5.02 ± 0.89€,¥ | 6.71 ± 2.75 | 14.32 ± 6.55 | 15.82 ± 7.49 | 12.29 ± 5.14 | 7.41 ± 3.12 |
| Heart Rate (bpm) | 462 ± 11.52 | 450.9 ± 17.83 | 476.6 ± 37.14 | 422.4 ± 68.37 | 511.7 ± 31.01 | 476.6 ± 38.13 |
| Ejection Fraction (%) | 53.47 ± 9.3 | 34.76 ± 10.25**,* | 53.89 ± 4.23 | 54.54 ± 4.15 | 56.37 ± 14.14 | 46.64 ± 8.90 |
| Ea (mmHg/μL) | 3.42 ± 0.28 | 6.15 ± 1.55***,**** | 3.58 ± 0.59 | 3.16 ± 0.95€€€ | 2.52 ± 0.71 | 2.47 ± 0.78¥¥¥¥ |
| Power Max (mmHg×μL/s) | 5654 ± 1973 | 4601 ± 2510 | 10409 ± 5482 | 6174 ± 2834 | 13567 ± 5462 | 6137 ± 2988* |
| dP/dt max (mmHg/s) | 6416 ± 660 | 7235 ± 727*, €€ | 5068 ± 469.5† | 4812 ± 1390 | 6818 ± 1446 | 6446 ± 661 |
| dP/dt min (mmHg/s) | -7292 ± 970.5 | -6978 ± 1925 | -4818 ± 1124 | -5342 ± 813.7 | -6643 ± 2519 | -6935 ± 1343 |
| dV/dt max (μL/s) | 605.9 ± 103.9 | 407.7 ± 148.8* | 606.3 ± 77.31 | 519.5 ± 86.13 | 1042 ± 213.7¥¥¥,††† | 758.4 ± 167.7*,*,¥¥ |
| dV/dt min (μL/s) | -800.4 ± 94.54 | -569.5 ± 138.9** | -681.7 ± 115.1 | -784.8 ± 212.3 | -1184 ± 350.4 | -961.1 ± 290.9 |
| P@dV/dt max (mmHg) | 2.85 ± 1.33¥,€ | 4.00 ± 2.14 | 9.06 ± 4.87 | 11.31 ± 5.01 | 9.79 ± 5.06 | 4.68 ± 2.8 |
| P@dP/dt max (mmHg) | 47.36 ± 12.6 | 55.98 ± 12.55 | 38.74 ± 6.12 | 38.09 ± 4.93 | 51.49 ± 11.89 | 42.06 ± 14.05 |
| V@dP/dt max (μL) | 48.56 ± 9.41 | 47.92 ± 10.32 | 45.4 ± 8.32 | 48.48 ± 8.44 | 71.47 ± 17.34¥¥,†† | 64.17 ± 6.51 |
| V@dP/dt min (μL) | 23.26 ± 8.18 | 31.78 ± 9.19 | 23.75 ± 6.26 | 22.93 ± 3.21 | 35.83 ± 17.32 | 36.71 ± 9.04 |
| Tau (ms) | 6.49 ± 0.62 | 7.72 ± 2.15 | 10.24 ± 2.91 | 11.03 ± 3.91 | 9.03 ± 2.28 | 6.56 ± 1.41 |
| dP/dt max (mmHg) occlusion | 100.8 ± 55.41 | 197 ± 65.87* | 35.47 ± 20.81 | 56.29 ± 16.45€ | 76.68 ± 45.34 | 99.06 ± 25.67 |
| PRSW | 70.66 ± 15.95 | 67.57 ± 12.53 | 39.53 ± 17.28 | 54.56 ± 4.95€ | 56.69 ± 17.71 | 64.33 ± 11.65 |

ESPVR: End-systolic Pressure-Volume Relationship, EDPVR: End-diastolic Pressure-Volume Relationship; Ea: Arterial Elastance; P: Pressure, t: Time, V: Volume; Max: Maximum; Min: Minimum; Tau: Time Constant of LV Relaxation. Values are mean ± SD. *p<0.05, **p<0.01, ***p<0.001 and ****p<0.0001 compared Col4a3-/- with their respective control (same strain), †p<0.05, ††p<0.01, †††p<0.001, ††††p<0.0001 differences between Balb/C strain and C57Bl/6 strain (WT Vs WT and Col4a3-/- vs Col4a3-/-), €p<0.05, €€p<0.01, €€€p<0.001, €€€€p<0.0001 differences between Balb/C strain and 129J (WT vs WT and Col4a3-/- vs Col4a3-/-), ¥P<0.05, ¥¥p<0.01, ¥¥¥ p<0.001, ¥¥¥¥p<0.0001 difference between 129J and C57Bl/6 (WT Vs WT and Col4a3-/- vs Col4a3-/-). Two *p<0.05, **p<0.01, ***p<0.001 and ****p<0.0001 compared Col4a3-/- with their respective control (same strain) using t-test (parameters with normal distribution) or Mann-Whitney test (for data following non normal distribution).

**Table S5.** Pressure-volume analyses and hemodynamic data of female mice from 129J, Balb/C and C57Bl/6 background by ventricular catheterization

| Echocardiography parameters | 129J 8 weeks old | | Balb/C 8 weeks old | | C57Bl/6 20 weeks old | |
|---|---|---|---|---|---|---|
| | **Pulmonary valve** | | | | | |
| | **WT (n=8)** | ***Col4a3**^{-/-}* **(n=9)** | **WT (n=6)** | ***Col4a3**^{-/-}* ***(n=6)*** | **WT (n=6)** | ***Col4a3**^{-/-}* ***(n=5)*** |
| ESPVR Slope | 2.88 ± 0.78[¥] | 3.07 ± 0.79[€] | 2.13 ± 0.62 | 1.7 ± 0.65 | 1.64 ± 0.53 | 1.2 ± 1.03 |
| EDPVR Slope | 0.12 ± 0.03 | 0.32 ± 0.20* | 0.13 ± 0.06 | 0.39 ± 0.26 | 0.16 ± 0.03 | 0.37 ± 0.16** |
| Stroke Work (mmHg×µL) | 1831 ± 465.9 | 1431 ± 538.2 | 1321 ± 412.7[€,†] | 1037 ± 173.9 | 2037 ± 241.7 | 1578 ± 104.8** |
| Cardiac Output (µL/min) | 9281 ± 2201[¥¥] | 7544 ± 2803[¥] | 9563 ± 2365[††] | 7961 ± 1565 | 14417 ± 2410 | 11417 ± 1015* |
| Stroke Volume (µL) | 22.25 ± 5.24 | 18.02 ± 5.44 | 21.2 ± 6.36 | 18.45 ± 2.49 | 30.41 ± 4.57[¥,†] | 23.63 ± 2.28** |
| Volume Maximum (µL) | 39.73 ± 5.76 | 39.86 ± 10.44 | 40.37 ± 11.79 | 37.66 ± 5.71 | 50.42 ± 5.79 | 52.81 ± 11.57 |
| Volume Minimum (µL) | 15.99 ± 5.39 | 20.73 ± 7.86 | 19.16 ± 6.92 | 19.2 ± 5.8 | 20 ± 2.15 | 29.18 ± 11.5 |
| End-systolic Volume (µL) | 17.89 ± 5.91 | 22.7 ± 7.94 | 23.08 ± 7.17 | 23.54 ± 6.76 | 24.73 ± 2.11 | 31.38 ± 11.35 |
| End-diastolic Volume (µL) | 37.4 ± 5.48 | 38.81 ± 9.94 | 39.44 ± 11.3 | 36.57 ± 5.41 | 49.45 ± 5.68 | 52.11 ± 11.84 |
| Pressure Maximum (mmHg) | 89.79 ± 5.11 | 89.43 ± 11.88[€€] | 79.94 ± 1.65 | 72.67 ± 8.37 | 91.6 ± 7.1 | 86.16 ± 4.0 |
| Pressure minimum (mmHg) | 1.99 ± 1.56[¥] | 3.48 ± 2.75 | 6.03 ± 1.8 | 6.4 ± 2.04 | 8.59 ± 6.3 | 6.83 ± 4.4 |
| Mean Pressure (mmHg) | 32.8 ± 2.3 | 36.01 ± 10.27 | 35.95 ± 4.73 | 34 ± 8.9 | 45.23 ± 11.36 | 39.51 ± 6.43 |
| Pressure Development (mmHg) | 87.8 ± 6.12[€] | 85.95 ± 10.77[€€] | 74.74 ± 4.1 | 66.26 ± 6.39* | 81.01 ± 2.14 | 79.34 ± 7.19 |
| End-systolic Pressure (mmHg) | 83.64 ± 5.9 | 84.06 ± 14.6 | 77.41 ± 3.8 | 68 ± 8.3*[,€] | 91.14 ± 7.42 | 78.76 ± 6.91* |
| End-diastolic Pressure (mmHg) | 4.24 ± 1.35[€] | 10.34 ± 4.62* | 9.79 ± 3.45 | 14.57 ± 9.55 | 16.38 ± 10.54 | 11.98 ± 5.22 |
| Heart Rate (bpm) | 418.9 ± 38.73 | 415 ± 73.26 | 457.6 ± 37.37 | 429.1 ± 33.93 | 473.3 ± 28.63 | 482.4 ± 19.06 |
| Ejection Fraction (%) | 57.81 ± 11.98 | 47.06 ± 12.13 | 54.29 ± 9.36 | 50.93 ± 9.51 | 63.41 ± 4.52 | 48.48 ± 13.85* |
| Ea (mmHg/µL) | 3.99 ± 1.13 | 5.05 ± 1.64 | 3.99 ± 1.41 | 3.71 ± 0.46 | 3.033 ± 0.28 | 3.35 ± 0.31 |
| Power Max (mmHg×µL/s) | 3539 ± 2866 | 5105 ± 4524 | 5476 ± 3080 | 5226 ± 3351 | 13117 ± 10621 | 8139 ± 2300 |
| dP/dt max (mmHg/s) | 6377 ± 543.4 | 5875 ± 15.07 | 5426 ± 904.1 | 3989 ± 489.6* | 6402 ±1100 | 6610 ± 1416[¥,††] |
| dP/dt min (mmHg/s) | -7453 ± 1205 | -6159 ± 1905 | -5414 ± 1348 | -4722 ± 511.3 | -5931 ± 1582 | -6402 ± 1531 |
| dV/dt max (µL/s) | 448.2 ± 157.1 | 461.6 ± 136.8 | 486 ± 110 | 459.8 ± 109.7 | 827.4 ± 262.3[¥¥,††] | 639.9 ± 106.6 |
| dV/dt min (µL/s) | -688 ± 179.9 | -567.3 ± 193 | -577.9 ± 219.6 | -505.2 ± 127.1 | -783.9 ± 95.49 | -695.7 ± 66.62 |

| | | | | | | |
|---|---|---|---|---|---|---|
| P@dV/dt max (mmHg) | 2.97 ± 1.49 | 4.60 ± 2.42 | 6.26 ± 1.79 | 6.6 ± 2.05 | 9.05 ± 6.16¥ | 7.20 ±4.45 |
| P@dP/dt max (mmHg) | 49.62 ± 4.78 | 49 ± 10.59 | 36.98 ±5.33€ | 33.82 ± 7.63€ | 51.34 ± 5.20 | 42.87 ±5.11* |
| V@dP/dt max (μL) | 38.55 ± 5.72 | 39.06 ± 10.03 | 39.28 ± 11.38 | 36.98 ± 6.29 | 47.89 ± 5.21 | 51.44 ± 11.91 |
| V@dP/dt min (μL) | 16.34 ± 5.57 | 21.24 ± 7.76 | 19.58 ± 6.75 | 19.38 ± 6† | 20.42 ± 2.23 | 33.07 ± 7.39***,*,¥ |
| Tau (ms) | 6.74 ± 0.73 | 9.33 ± 2.31** | 9.77 ± 3.82 | 8.98 ±1.23 | 9.35 ±3.23 | 8.3 ± 2.8 |
| dP/dt max (mmHg) occlusion | 88.51 ± 45.03 | 112.6 ± 54.09 | 95.3 ± 54.63 | 55.01 ±15.82 | 90.83 ± 38.41 | 93.62 ± 28.92 |
| PRSW | 60.86 ± 10.03 | 57.08 ± 11.15 | 47.93 ± 6.81 | 38.06 ± 11.33 | 42.42 ± 16.18 | 42.54 ± 22.4 |

ESPVR: End-systolic Pressure-Volume Relationship, EDPVR: End-diastolic Pressure-Volume Relationship; Ea: Arterial Elastance; P: Pressure, t: Time, V: Volume; Max: Maximum; Min: Minimum; Tau: Time Constant of LV Relaxation. Values are mean ± SD. *p<0.05, **p<0.01, ***p<0.001 and ****p<0.0001 compared Col4a3-/- with their respective control (same strain), †p<0.05, ††p<0.01, †††p<0.001, ††††p<0.0001 differences between Balb/C strain and C57Bl/6 strain (WT vs WT and Col4a3-/- vs Col4a3-/-), €p<0.05, €€p<0.01, €€€p<0.001, €€€€p<0.0001 differences between Balb/C strain and 129J (WT vs WT and Col4a3-/- vs Col4a3-/-), ¥P<0.05, ¥¥p<0.01, ¥¥¥ p<0.001, ¥¥¥¥p<0.0001 difference between 129J and C57Bl/6 (WT Vs WT and Col4a3-/- vs Col4a3-/-). Two *p<0.05, **p<0.01, ***p<0.001 and ****p<0.0001 compared Col4a3-/- with their respective control (same strain) using t-test (parameters with normal distribution) or Mann-Whitney test (for data following non normal distribution).
